# Supplementary figures and images for: Adipocyte NR1D1 dictates adipose tissue expansion during obesity
Source: eLife. 2021 Aug 5;10:e63324. doi: 10.7554/eLife.63324 (PMC8360653; doi:10.7554/eLife.63324)

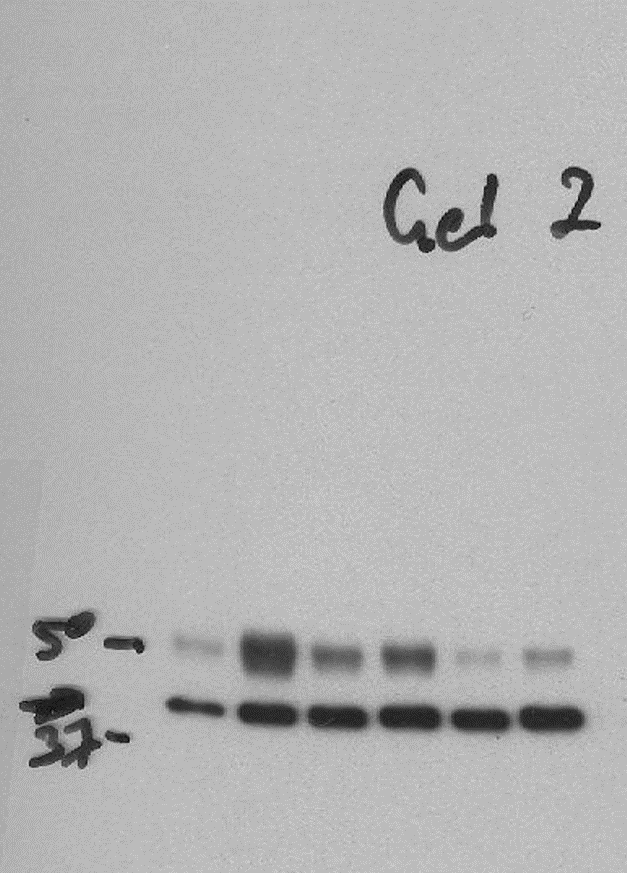

Supplement: Figure 1—figure supplement 1—source data 1. [file elife-63324-fig1-figsupp1-data1.zip › Figure 1-figure supplement 1-source data 1/ACTB.png]

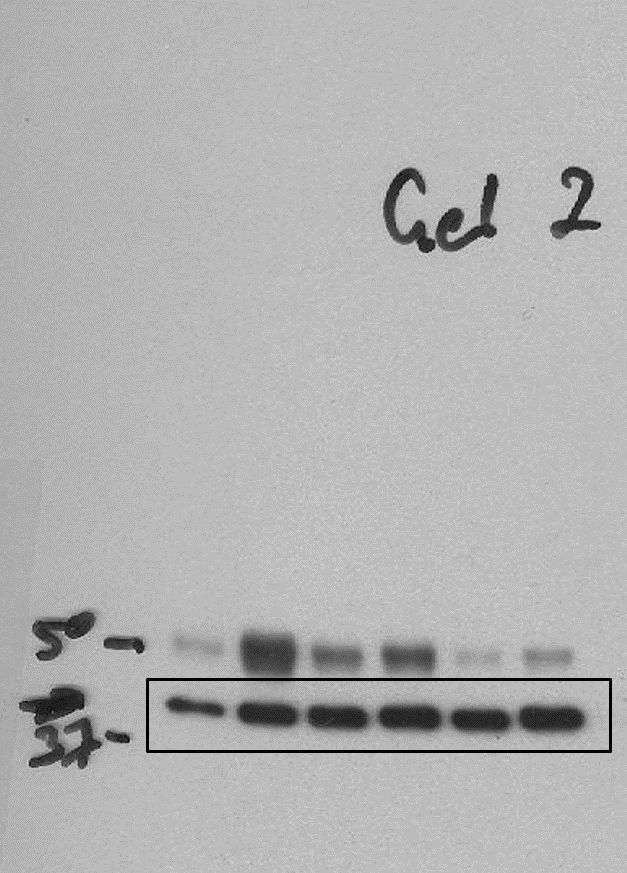

Supplement: Figure 1—figure supplement 1—source data 1. [file elife-63324-fig1-figsupp1-data1.zip › Figure 1-figure supplement 1-source data 1/ACTB_bands_indicated.png]

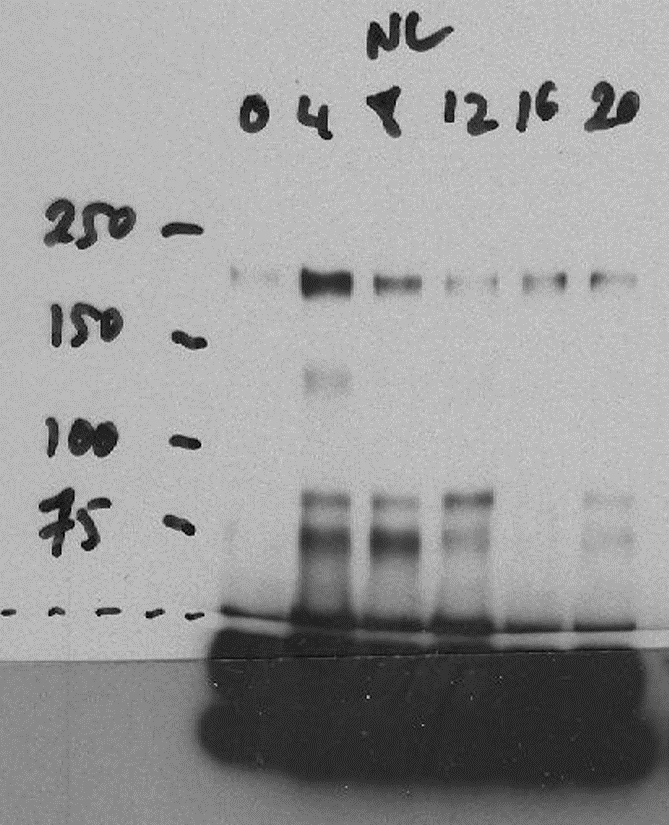

Supplement: Figure 1—figure supplement 1—source data 1. [file elife-63324-fig1-figsupp1-data1.zip › Figure 1-figure supplement 1-source data 1/NR1D1.png]

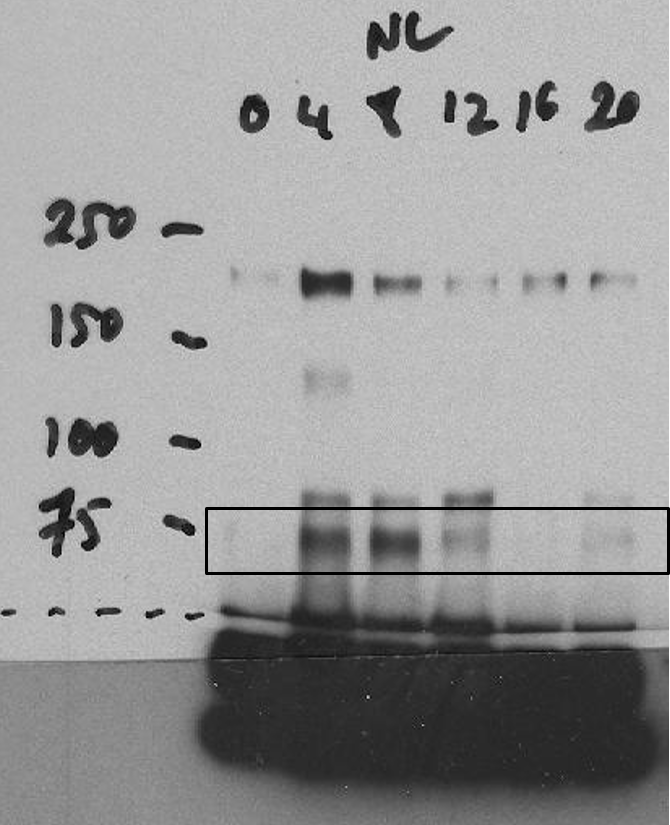

Supplement: Figure 1—figure supplement 1—source data 1. [file elife-63324-fig1-figsupp1-data1.zip › Figure 1-figure supplement 1-source data 1/NR1D1_bands_indicated.png]

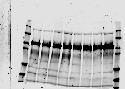

Supplement: Figure 2—source data 1. [file elife-63324-fig2-data1.zip › Figure 2-source data 1/NR1D1 raw files/0002157_01_TH.jpg]

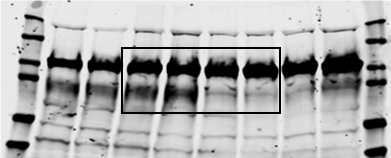

Supplement: Figure 2—source data 1. [file elife-63324-fig2-data1.zip › Figure 2-source data 1/NR1D1_bands_indicated.png]

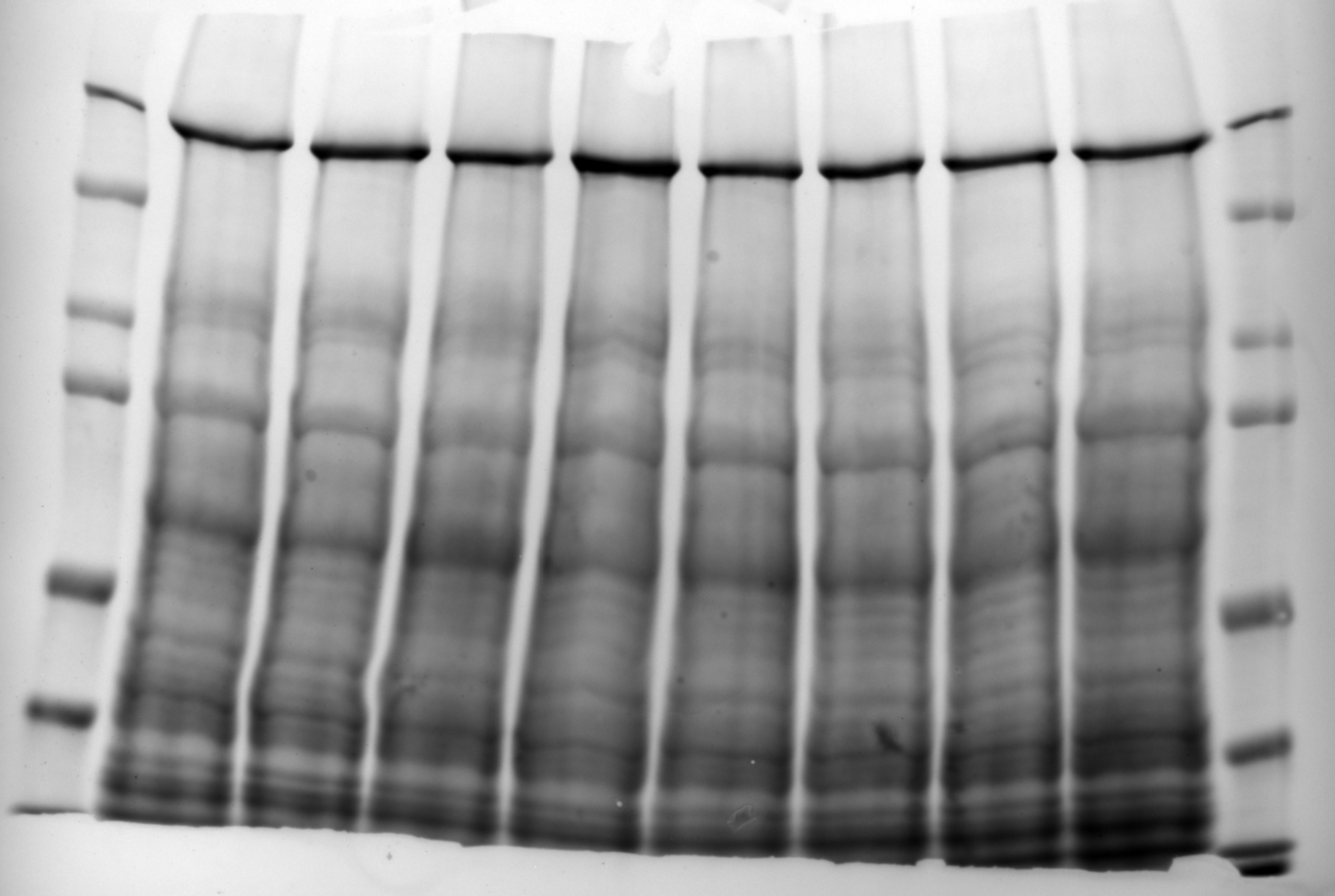

Supplement: Figure 2—source data 1. [file elife-63324-fig2-data1.zip › Figure 2-source data 1/Ponceau.tif]

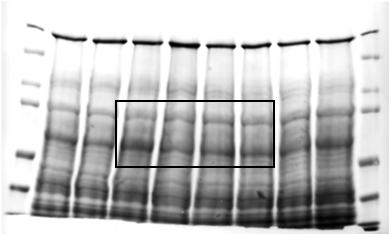

Supplement: Figure 2—source data 1. [file elife-63324-fig2-data1.zip › Figure 2-source data 1/Ponceau_bands_indicated.png]

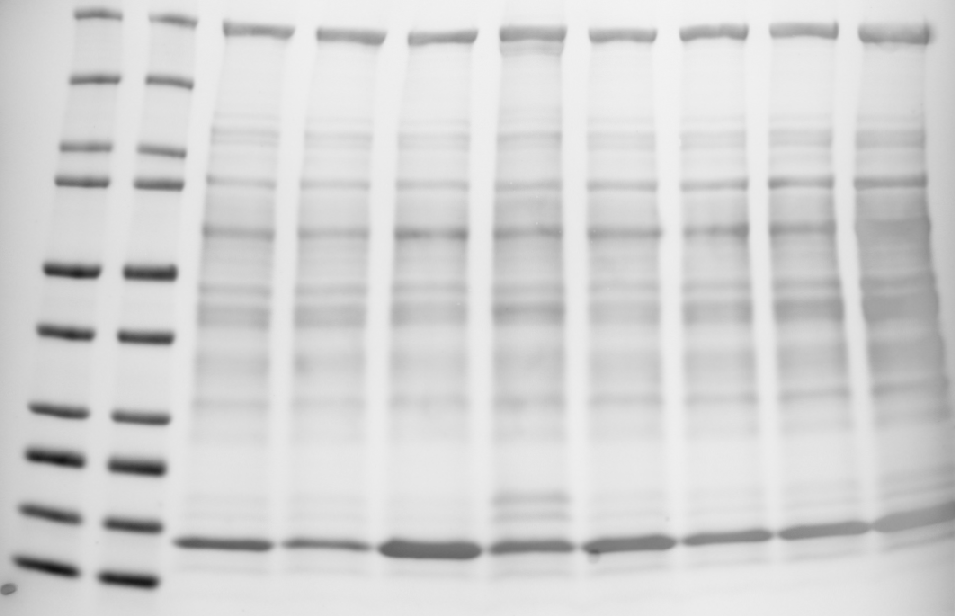

Supplement: Figure 2—figure supplement 1—source data 1. [file elife-63324-fig2-figsupp1-data1.zip › Figure 2-figure supplement 1-source data 1/Ponceau.tif]

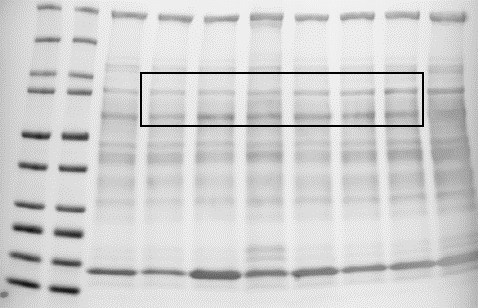

Supplement: Figure 2—figure supplement 1—source data 1. [file elife-63324-fig2-figsupp1-data1.zip › Figure 2-figure supplement 1-source data 1/Ponceau_bands_indicated.png]

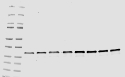

Supplement: Figure 2—figure supplement 1—source data 1. [file elife-63324-fig2-figsupp1-data1.zip › Figure 2-figure supplement 1-source data 1/UCP1 raw files/0000638_01_TH.jpg]

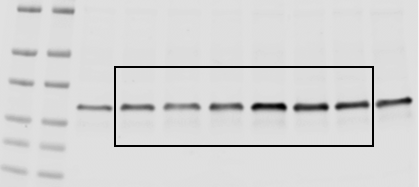

Supplement: Figure 2—figure supplement 1—source data 1. [file elife-63324-fig2-figsupp1-data1.zip › Figure 2-figure supplement 1-source data 1/UCP1_bands_indicated.png]
