# Supplementary material for: Adipocyte NR1D1 dictates adipose tissue expansion during obesity
Source: eLife. 2021 Aug 5;10:e63324. doi: 10.7554/eLife.63324 (PMC8360653; doi:10.7554/eLife.63324)

Nr1d1$^{Flox2\text{-}6}$: Normal chow (252, 279, 280, 288)

x10 magnification (bright field and polarised light); 200µm scale bars

252

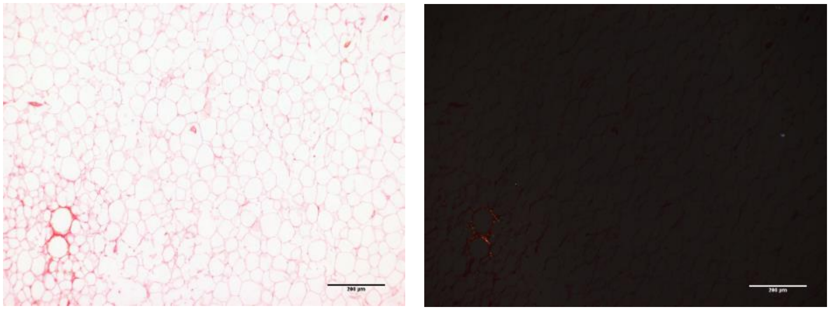

279

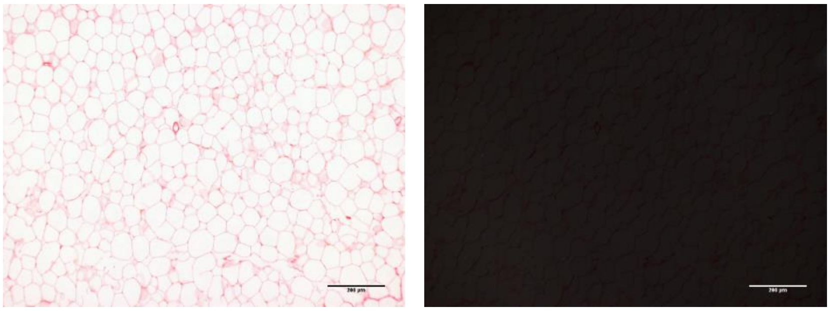

280

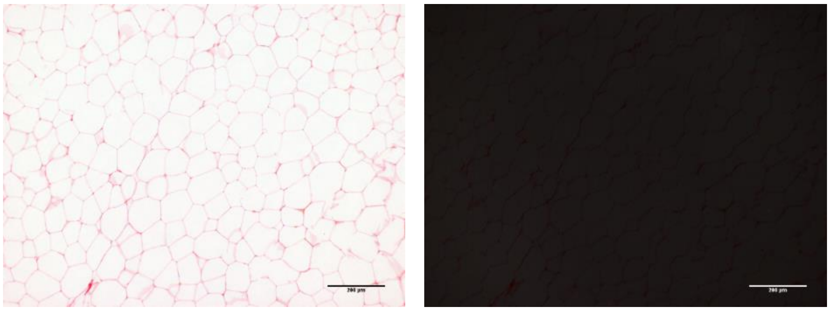

288

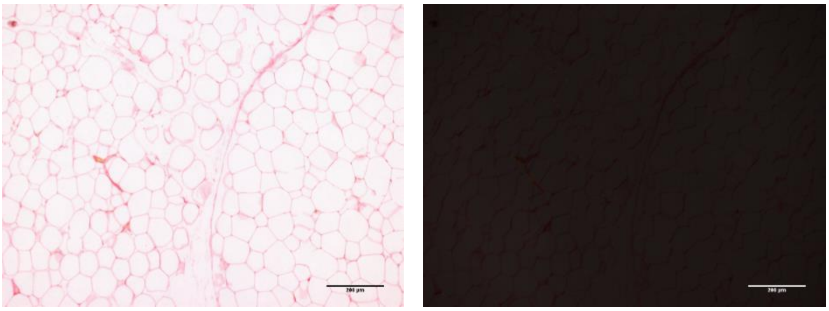

Nr1d1$^{Flox2\text{-}6}$: High Fat Diet (188, 189, 194, 243, 248, 246)

188

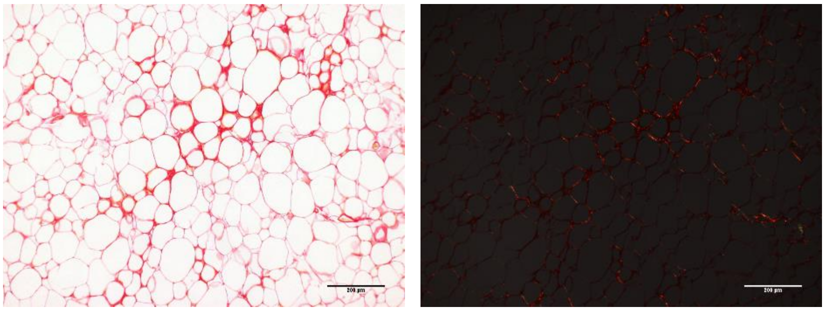

189

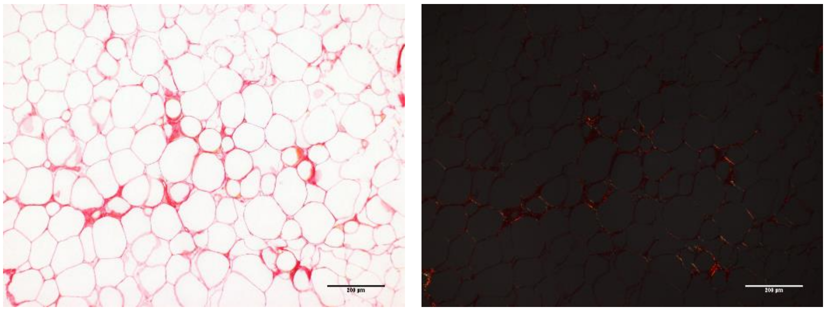

194

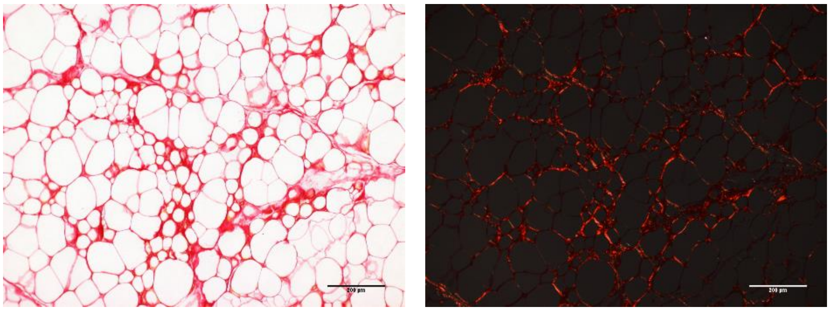

243

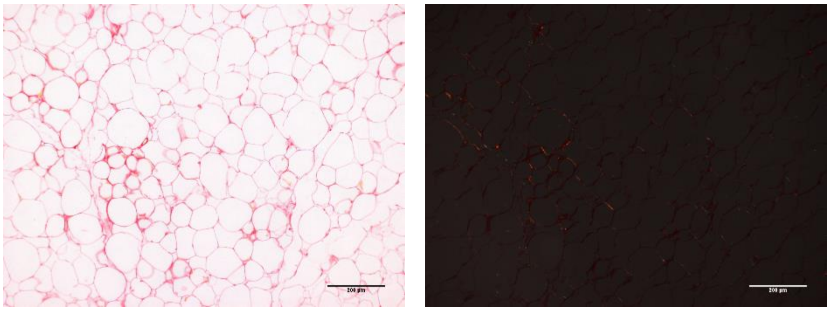

248

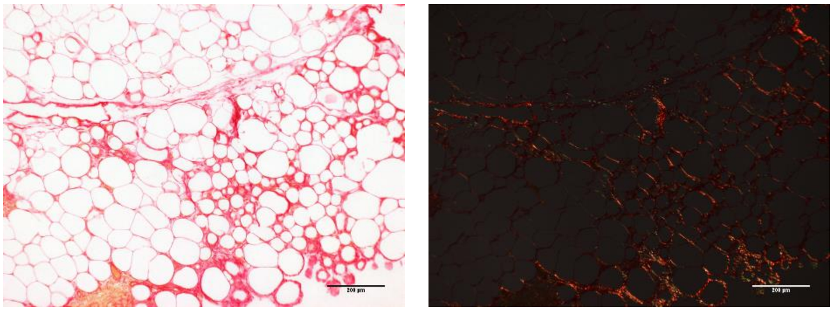

246

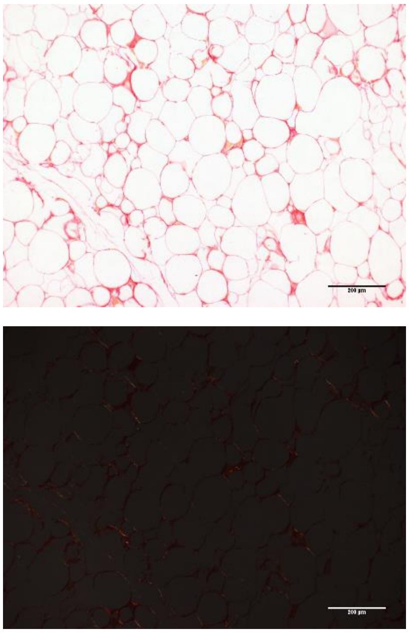

Nr1d1$^{Flox2\text{-}6}$:*Adipoq*^{Cre} : Normal chow (250, 251, 278, 281)

250

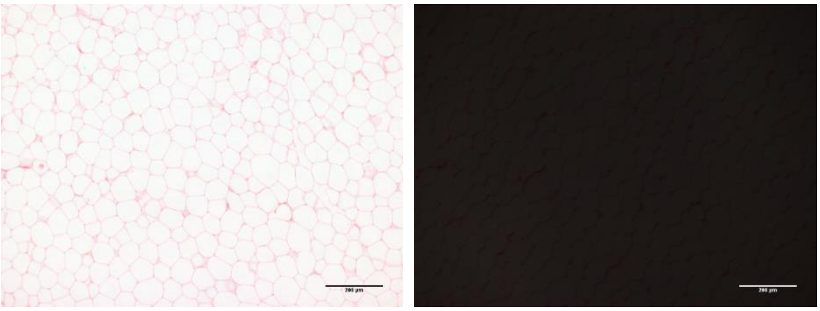

251

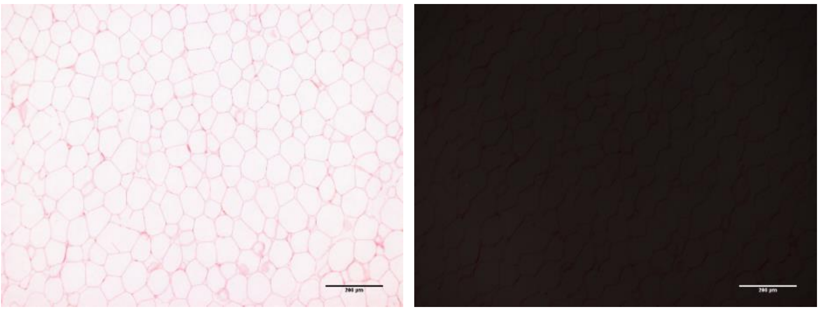

278

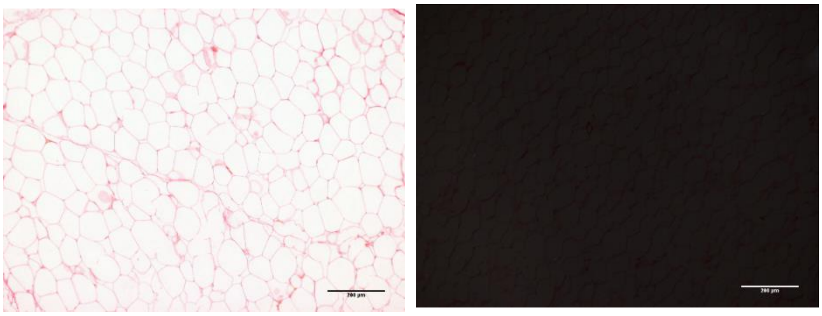

281

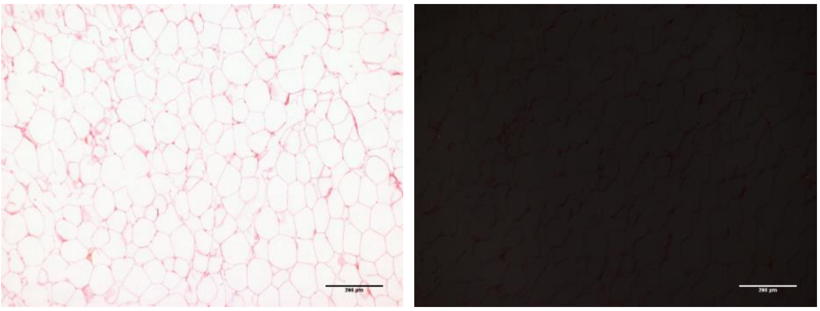

Nr1d1$^{Flox2-6}$:*Adipoq*^{Cre} : High Fat Diet (195, 196, 197, 238, 240, 239)

195

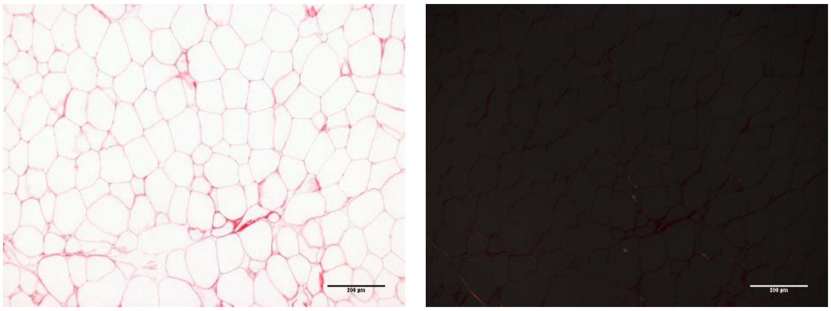

196

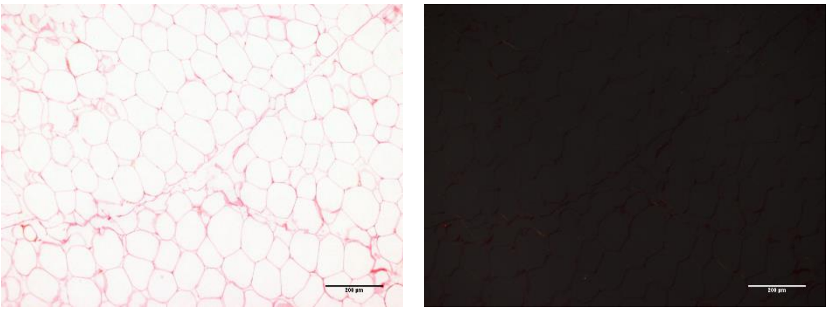

197

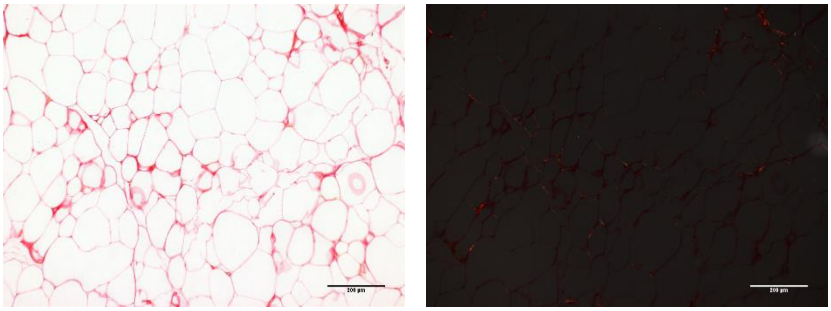

238

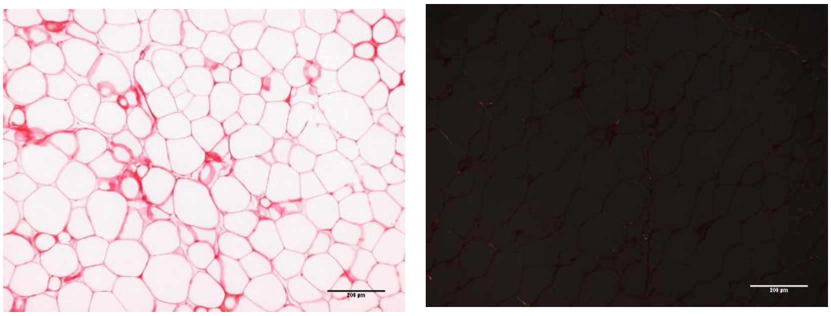

240

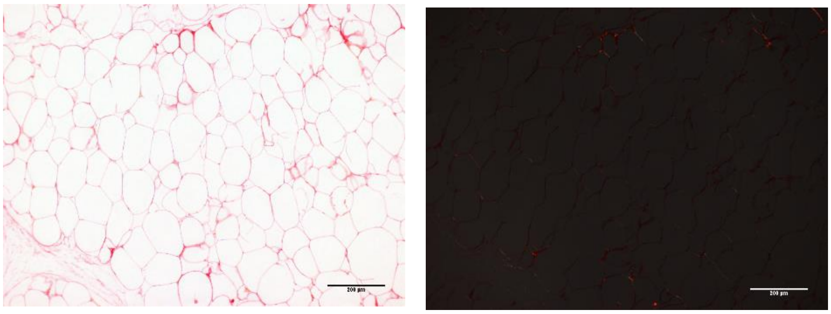

239

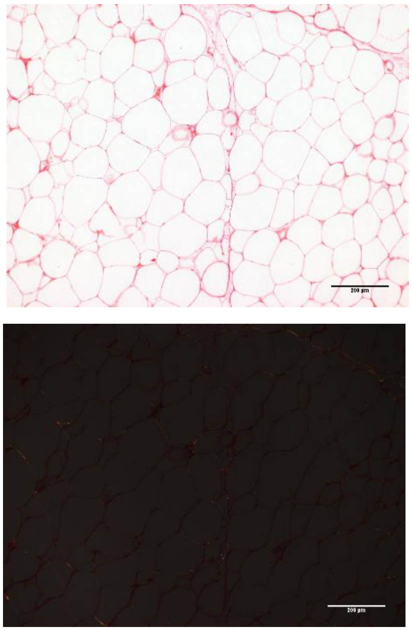

Supplement: Figure 4—source data 1. [file elife-63324-fig4-data1.pdf]
